# Impacts of environmental stressors on fertility and fecundity across taxa, with implications for planetary health

**DOI:** 10.1038/s44454-026-00032-6

**Published:** 2026-04-23

**Authors:** Susanne M. Brander, Shanna H. Swan, Alvine C. Mehinto, Karen A. Kidd, Judith S. Weis, Scott M. Belcher, Jamie C. DeWitt, Stacey L. Harper, Caren C. Helbing

**Affiliations:** 1 Coastal Oregon Marine Experiment Station, College of Agricultural Sciences, Oregon State University, Newport, OR USA; 2 Department of Environmental Medicine, Icahn School of Medicine at Mount Sinai, New York, NY USA; 3 Department of Toxicology, Southern California Coastal Water Research Project, Costa Mesa, CA USA; 4 Department of Biology, McMaster University, Hamilton, ON Canada; 5 Department of Biological Sciences, Rutgers University, Newark, NJ USA; 6 Department of Biological Sciences, North Carolina State University, Raleigh, NC USA; 7 Department of Environmental and Molecular Toxicology, Oregon State University, Corvallis, OR USA; 8 Department of Environmental and Molecular Toxicology & School of Chemical, Biological and Environmental Engineering, Oregon State University, Corvallis, OR USA; 9 Department of Biochemistry and Microbiology, University of Victoria, Victoria, BC Canada

**Keywords:** Ecology, Ecology, Environmental sciences

## Abstract

Exposure to synthetic chemicals occurs across species. These substances are often untested, highly persistent, and lack regulation. Together with climate change, they can cause population decline. Many act as endocrine-disrupting chemicals, interfering with hormones at low concentrations. Emerging pollutants, including microplastics and per- and polyfluoroalkyl substances, further contribute. Impacts include reduced fertility, fecundity, and even multigenerational harm. Cross-species evidence underscores the need for systemic approaches to protect biodiversity and planetary health.

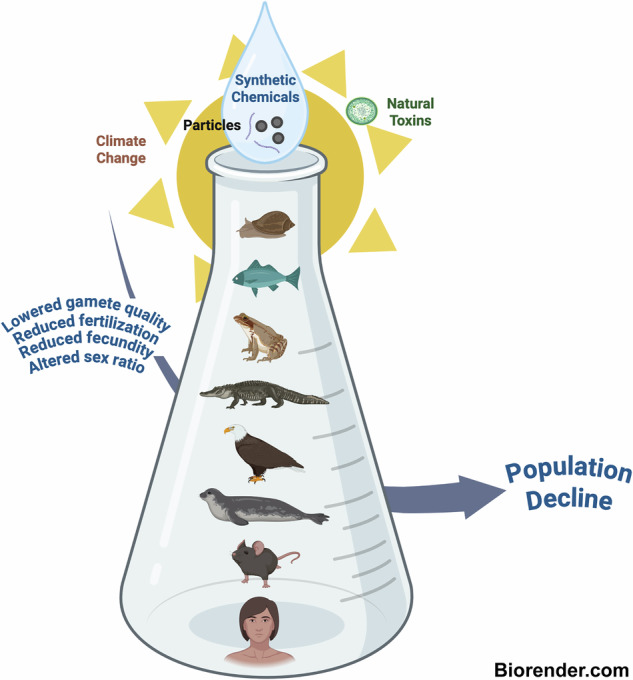

## Introduction

Humans, fish, and wildlife are constantly ingesting, absorbing, and inhaling synthetic chemicals (“novel entities”), which are far from benign, have not been thoroughly tested, and can lack the regulatory frameworks necessary to determine safety thresholds^1–3^. Additionally, all living organisms are exposed to a wide variety of environmental stressors simultaneously, including warming temperatures and associated increases in toxic algal blooms or otherwise altered abiotic conditions^4^. These combined persistent “novel entities,” sometimes referred to as the chemical exposome^5^, often outlive their utility by decades or centuries, and together with climate change threaten to exceed planetary boundaries^6^. Meanwhile, more than 2000 new chemicals are introduced globally each year^7^.

Of >140,000 registered synthetic chemicals under REACH, over 1000 are recognized as endocrine disrupting chemicals (EDCs)^8^. However, this is likely a gross underestimate, given that only 1% of synthetic chemicals have been sufficiently evaluated for safety^9^. EDCs act by mimicking or blocking hormones and altering their functions across taxa. They can bind to hormone receptors, alter enzyme activities, and/or modify epigenetic signals, thereby triggering harmful effects across taxonomic groups,. This can occur at effective concentrations so low they are analogous to a whisper that is powerful enough to redirect a hurricane^10^. EDC dose-response curves are often non-linear, meaning effects can occur at low levels, and those same effects may not occur at higher exposures. Research on estrogenic chemicals such as bisphenol A (BPA) and anti-androgenic chemicals such as phthalates—plasticizers that leach into foods and waterways—are a few examples of numerous chemicals linked to similar reproductive problems across fish, wildlife, and humans. Yet these chemicals remain widely used^11^.

**Fig. 1:**
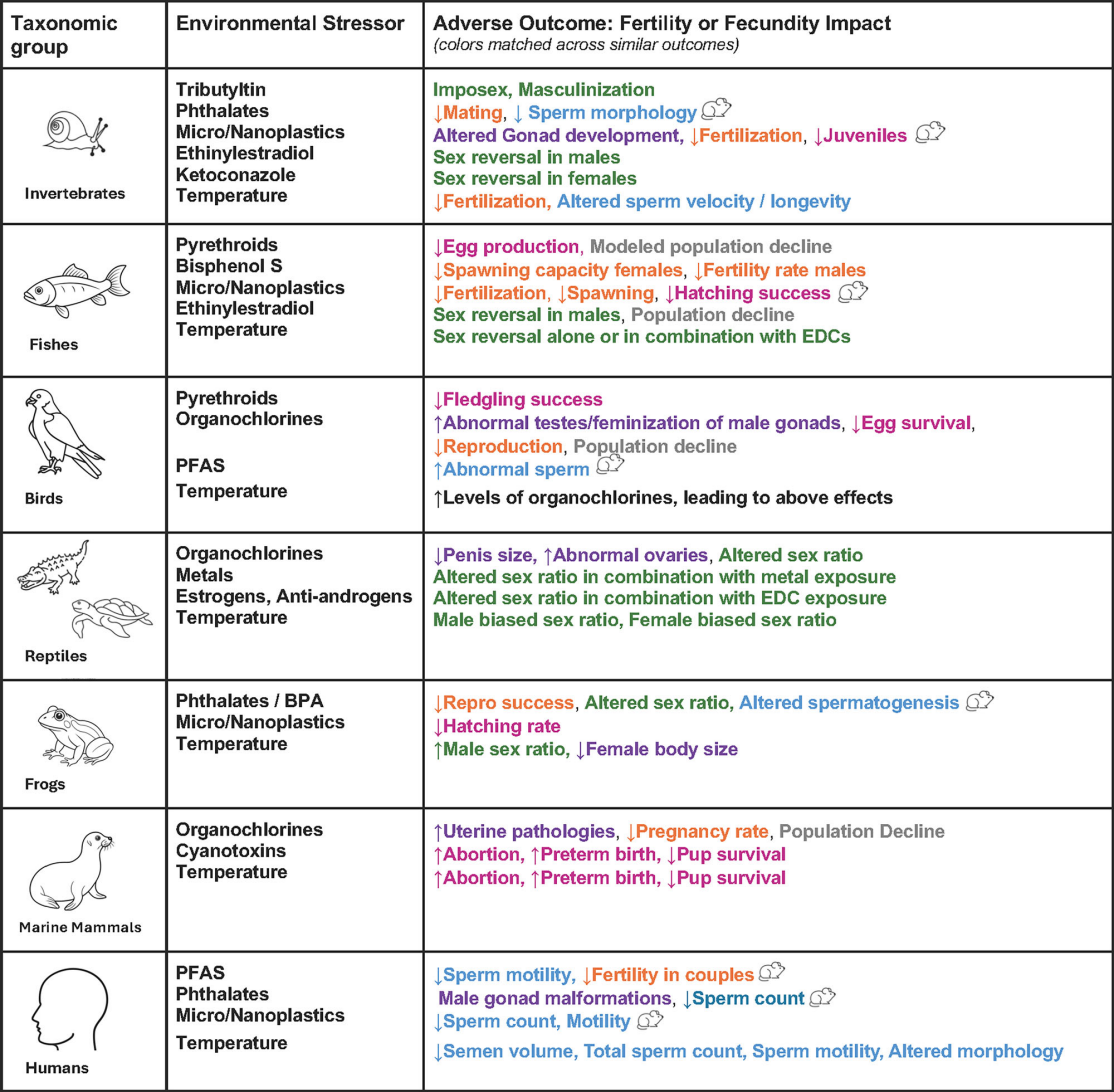
Summary of reviewed responses with relevance to fertility and/or fecundity across taxonomic groups. Similar endpoints among contaminants and groups are highlighted in the same color. Adverse outcomes also observed in rodents are indicated by a rodent icon. Images are credited to The Noun Project.

To complicate matters further, negative influences on reproduction are not limited to traditional EDCs. Additional classes of emerging pollutants, along with climate-related factors such as warming temperatures and associated decreases in oxygen levels, are now documented to also cause reproductive declines^12–15^. For example, it is already known that temperature plays a role in sex determination in fish, reptiles, and amphibians, that EDCs can alter environmental sex determination, and that heat-related stress can exacerbate conditions such as infertility in humans^16,17^. Furthermore, pollutants not yet classified as reproductive toxicants, such as microplastics, have been found in placentas and eggs^18,19^. These particles may potentially lower fertility through translocation and associated oxidative damage to cells and tissues, which can in turn alter steroidogenesis and downstream responses to reproductive hormones^20–22^. Per- and polyfluoroalkyl substances (PFAS), which also can disrupt reproductive hormone signaling in a myriad of ways, are still under intensive study, are used in products from raincoats to food wrappers, and are commonly discharged to waterways and drinking water sources^14,23^. Although older PFAS like perfluorooctanoic acid (PFOA) have been mostly phased out, newer versions can produce similar toxicological outcomes. Chemical stressors combined with warming temperatures are demonstrated to often have additive or synergistic effects^13,24^. While scientists have long known that steroidogenic chemicals affect reproductive aspects of the endocrine system, consideration of pollutants that act via unknown mechanisms, and the outsized influence of climate-related factors is crucial if we are to understand the combined and cumulative effects of exposure (Fig. 1).

Reduced fecundity and fertility caused by cumulative (and multi-generational) exposure to these toxicants is, perhaps, their most concerning impact^25^. The term fertility is primarily used to refer to humans, but across species can be defined as the number of viable offspring produced (usually in a specified time frame). Fecundity is used to describe the biological capacity to reproduce, which can include egg production, gamete quality, and other factors. Observations of reduced fertility, hatchability, and egg production following toxic exposure have been demonstrated in fruit flies as well as a variety of aquatic invertebrates^13,26,27^. Similarly, in vertebrates, responses include: intersex individuals (e.g., in fish), incomplete masculinization, reduced egg production, and, ultimately, population decline^28–31^. These responses likely contribute to reduced global biodiversity, which is inherently linked to the population decline of species sensitive to both pollution and other stressors^32,33^.

**Fig. 2:**
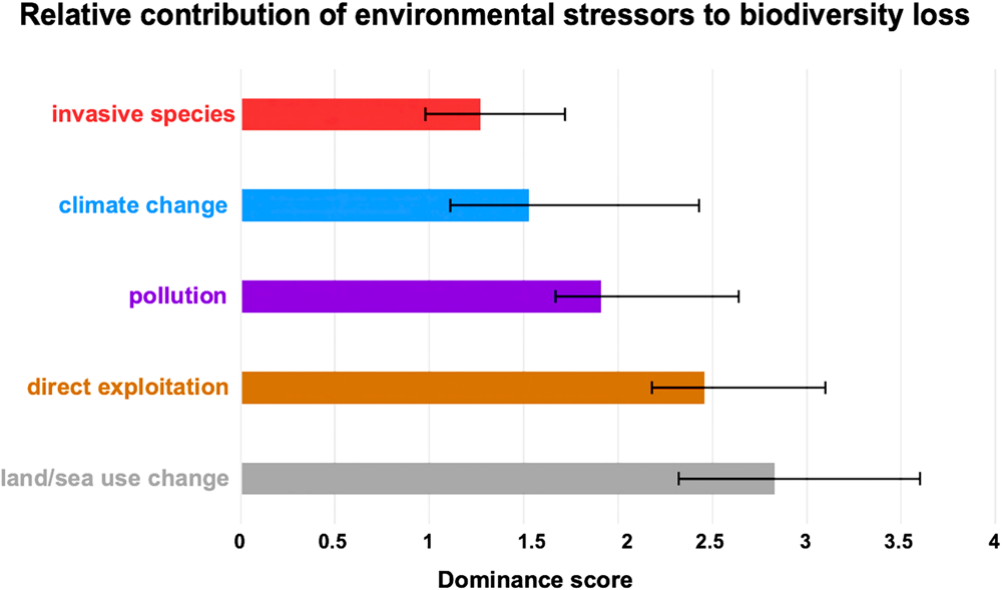
Adapted from data described in ref. ^33^ Extracted information from 163 studies that included nonredundant comparisons of the impacts on biodiversity of at least two of the five predefined classes of direct drivers: climate change, land/sea use change, direct exploitation of natural resources, pollution, and invasive alien species. Data from each multi-driver assessment were converted into one or more nonredundant pairwise comparisons between drivers, and bootstrapping was used to test whether pairs of drivers differed significantly in their impact. Estimates vary based on whether they were measured on land, at sea, or in freshwater, as well as by geographical location. Added together, pollution and climate change have a larger impact than any single driver.

A recent analysis concluded that pollutants and climate change combined are the largest cause of biodiversity decline^33^ (Fig. 2). Ultimately, none of these exposures occur in isolation, and thus it is critical to consider the impacts of climate change alongside increased exposure to a variety of chemicals that can interfere with reproduction, and hence fecundity and fertility rates. Chemical exposure is occurring in the context of these global fluctuations in temperature and oxygen levels, exacerbating stress responses that are detrimental to organismal health and interfering with adaptations and strategies that help stabilize populations. To build a sustainable future, we must recognize that chemicals, once released, don’t simply disappear. Instead, they contribute to the larger issue of driving humanity towards the exceedance of planetary boundaries when considered in combination with climate change and other planetary-level impacts^6^. Here, we review selected case studies across taxonomic groups from invertebrates to humans, chosen by experts in the field, demonstrating that these chemical and abiotic stressors act in concert to reduce population size and hence biodiversity across the entire phylogenetic tree.

### Invertebrates

Long before the term “endocrine disruptors” was coined by Theo Colborn^34^, chemical-induced reproductive problems in snails in the early 1980s led to the banning of a particular chemical as an antifouling agent by much of the world. Females of the American mud snail (*Nassarius obsoletus*) living in estuarine areas with marinas had developed a condition termed “imposex,” in which male structures (penis and vas deferens) developed in females^35,36^. Testing of other chemicals present in marinas showed that only tributyltin (TBT) compounds or paints induced imposex. However, exposure didn’t appear to reduce snail reproduction.

Studies in many areas in Southeast England of the predatory snail dog whelk, *Nucella lapidus*, found that populations close to boating and shipping activities were in decline^37^. Females were fewer in number and dominated by older, infertile individuals with intermediate and late stage imposex. The early stage involves the formation of the vas deferens and a small penis, the intermediate stage involves penis enlargement, and the late-stage oviduct occlusion. Late imposex females may contain incompletely formed or aborted capsules (often containing eggs) not released because of oviduct blockage. There was little or no evidence of breeding. The severity of imposex was related to the population’s proximity to a source of TBT (boats). Laboratory TBT exposure (0.02 μg/L) showed imposex progressed from early to late stage in six months^37^.

Follow-up studies found that transplanting *N. lapillus* from a ‘ clean’ locality with little boating to one close to a marina caused a major increase in the degree of imposex; analyses revealed that they bioaccumulated tributyltin^38^. Lab exposures confirmed this. Studies found that many other snail species responded the same way^39^. Subsequently, it was determined that the development of imposex was mediated by increasing androgen levels in the female snails and inhibited by increasing their estrogen^40^. These findings demonstrating that tributyltin was harming nontarget organisms prompted restrictions on its use in several European and other countries in the early 1980s. After warnings by US scientists at congressional hearings, Congress enacted restrictive legislation in 1988. This law resulted from unusually rapid congressional action and circumvention of usual USEPA regulatory procedures^41–43^ An outright ban is exceedingly rare, but it led to a major reduction of imposex in gastropods nationwide.

In addition to the clear-cut case of imposex caused by tributyltin, effects of phthalates, a common plasticizer, and other estrogenic chemicals have also been demonstrated in invertebrates. Dimethyl phthalate (DMP) in *Potamopyrgus antipodarum*, a sensitive snail species, causes differences in mating behavior and sperm morphology, which were primarily observed between controls and the high (10^−6^ M) DMP concentration group^44^. Mating frequency ultimately decreased by more than 69% with increasing DMP levels, and sperm morphology was increasingly altered relative to control males. At the intermediate and high concentrations, sperm tail length was significantly reduced, reflecting reduced fecundity.

Several studies have shown that bivalve mollusks also respond to vertebrate-like steroids. Evanson et al.^45^ evaluated the sex of *Mytilus edulis* using PCR prior to treatment and then exposed males to 17α-ethynylestradiol and females to androgenic ketoconazole. Both chemicals caused sex changes in treated mussels, interfered with gonadal development, and disrupted gene expression of the sex differentiation pathway. This confirmed a role for steroid-like hormones in mollusks, the mechanisms of which remain poorly understood.

Microplastics and nanoplastics (MNPs) can also disrupt the endocrine systems of invertebrates. For example, high-density polyethylene (PE) MNPs at environmental levels (0.0025 mg/L, for 14 days), in combination with DEHP, a phthalate plasticizer, impaired the reproductive capacity of female mussels (*Mytilus coruscus*) by suppressing estrogen receptors, cytochrome P450-3 (CYP3), and 17β-hydroxysteroid dehydrogenase (17β-HSD) gene expression^46^. These biochemical changes inhibited ovarian development and disrupted oocyte morphology. Additionally, MNPs have effects on corals. Wilkins et al.^47^ exposed *Montipora capitata* gamete bundles to virgin microspheres (nylon, polypropylene, high-density or low-density PE at 50, 100, or 200 particles/L) or leachates, presumably including plastic additives from these microspheres. Fertilization was not impaired by the particles themselves, but some leachates significantly reduced fertilization rates. Thus, plastic additives could pose an additional threat to coral persistence in coral reef ecosystems. These impacts are also observed in crustaceans, but with particles alone rather than in combination with associated chemicals. Similarly, polystyrene nanoplastics altered gene expression, hormone levels, and gonad development in juvenile river prawns (*Macrobrachium nipponense*)^48^. These observations align with the suspicion that MNPs disrupt reproductive functions across taxa.

Bio-based plastics, which are being touted as a replacement for the traditional polymers discussed above, are also not without issue. Guttierez-Rial et al.^49^ studied the effects of the bioplastics polylactic acid (PLA) and polyhydroxybutyrate (PHB) and conventional PE microplastics on the reproduction of the earthworm *Eisenia andrei*. Reproductive effects, including a decrease in cocoon production and the number of juveniles, were found after exposure to PE and PLA for 49 and 112 days, indicating that the reproductive toxicity of PLA was comparable to that of conventional plastic PE. This concern has now been raised across taxonomic groups, in terms of the potential toxicity of bio-based plastics and the risk of regrettable substitutions.

In summary, these diverse stressors can disrupt reproductive development and hormone pathways across mollusks, corals, crustaceans, and annelids, highlighting the broad ecological risks of plastic-associated contaminants.

### Fish

Like other organisms and most similar to other vertebrates., the endocrine system of fishes is highly sensitive and can be modulated by extremely low concentrations of endocrine active chemicals, especially when these molecules interact with specific hormone receptors (e.g., estrogen, androgen, glucocorticoid)^28,50–53^. Fishes are particularly vulnerable in terms of exposures as their habitats receive complex mixtures of treated effluent, sewage overflow, urban and agricultural run-off, and atmospheric deposition, all of which can contain components that disrupt hormone signaling. Impacts on fish are often used to infer potential adverse responses in higher vertebrates, and for this reason, fish are common model organisms used to better understand the overall effects of endocrine-active stressors^54^. Because the endocrine system is highly conserved across vertebrates, observations made regarding the impacts of chemicals and other stressors that may reduce fecundity and fertility in fishes may be cause for concern across organisms. Evidence points to this phenomenon across vertebrate species, highlighting the overall trend towards reduced fertility due to chemical exposure^55,56^. This has similarly been observed for climate change-induced stress from factors such as increased temperature and hypoxia^24^, both of which can increase the proportion of male fish in some species^12,30^.

Endocrine-active pesticides have been a challenge for decades, beginning with legacy organochlorines (e.g., DDT). Current-use classes pose increasing challenges to fish, especially as pesticide use increases due to climate change^24^. As it does in mammals, the organochlorine DDT causes altered hormone levels and reduced fertility, but due to the more flexible nature of sex determination in fishes, it can also cause feminization of males and subsequent alterations in sex ratios^57,58^. Newer classes of pesticides, such as pyrethroids, cause similar impacts across vertebrates, including fish^59^. For example, the pyrethroid bifenthrin is now one of the ten most commonly used pesticides in the U.S. Concentrations ranging from ng/L to low μg/L are frequently detected in waterways, particularly after rainfall in the vicinity of urban areas, where bifenthrin is often used to treat homes and lawns^60,61^. Research conducted with the euryhaline fish *Menidia beryllina* (Inland Silverside), which is widely distributed, highlights an adverse outcome pathway beginning with estrogen receptor modulation, which leads to egg protein decline, reduced fecundity, altered sex ratio depending on timing of exposure and other abiotic factors, and ultimately modeled declines in populations^7,29^. Exposure to bifenthrin and other EDCs is exacerbated by temperature change^62^. Similar endocrine-related impacts have been observed in zebrafish, rainbow trout, and mosquitofish, as well as rodent models, suggesting a common mechanism of action^59,63–65^.

Molecular initiating events involving alteration of estrogen-dependent gene and protein expression via receptor binding or alteration of steroidogenic enzyme activity are well documented for a number of chemical classes. However, as additional data become available for newer emerging contaminants such as MNPs, it is apparent that reproduction in vertebrates may be disrupted via less specific and still undefined pathways^20,66,67^. While more research is needed on the mechanisms by which MNPs interfere with reproductive capacity, oxidative stress and inflammation caused by the presence of foreign particles are emerging as a potential cause of reduced fecundity, possibly via disruption of the blood-testis barrier or oxidative imbalance in the ovaries, for example^21,68^. In adult female zebrafish, individuals were exposed to polystyrene microplastics, and effects on sirtuins, a family of NAD+ dependent proteins involved in the regulation of metabolism, gene expression, and oxidative stress, were examined in the context of their impact on reproduction^67^. Sirtuin SIRT1 specifically plays an important role in oogenesis and steroidogenesis. Exposure to polystyrene microplastics (MPs) (500 μg/L) caused reductions in fecundity, spawning, fertilization, and hatching success, and altered the development of the ovaries. These effects aligned with increased reactive oxygen species and reduced antioxidants, and disrupted hormone levels. Such impacts suggest that oxidative stress disrupts activities in the ovaries that then change hormone production, which can alter the entire hypothalamic pituitary gonadal axis. It has been suggested that these reproductive impacts can extend across generations in fishes^66^ and are exacerbated due to increased temperature, at least in terms of plastic additives such as BPA^69^. More research is needed on environmentally relevant plastic morphologies, in particular, commonly detected microfibers.

In summary, there is a large body of work demonstrating that exposure to classical endocrine disruptors and less studied stressors related to climate change and particle toxicity can modify fish reproductive capacity and, in some cases, cause reductions in population size^7,28,30,56^. While mechanisms of effects may differ across chemical classes and stressor types, the ultimate result is decreased fecundity, and the potential for biodiversity loss.

### Birds

As birds often occupy top trophic levels in terrestrial and aquatic food webs, their tissues accumulate elevated levels of diverse chemicals with known or suspected endocrine system impacts. As examples, polybrominated diphenyl ethers (PBDEs^70^), PFAS^71^, DDT^72^, and mercury (Hg^73^) are found globally in bird tissues, and lab exposures have linked them and other widespread contaminants (e.g., polychlorinated biphenyls (PCBs)) to declines in hormone levels and reproductive endpoints, including clutch sizes^74,75^. The following describes two case studies linking exposures of birds to synthetic chemicals with demonstrated or potential negative impacts on populations.

The decades-long and global use of the organochlorine insecticide DDT provides an unequivocal example of endocrine disruption leading to population-level effects in birds, especially in raptors, given DDT’s propensity to biomagnify through the food web, leading to elevated dietary exposures. Long-term monitoring data have shown declines in clutch and brood sizes, as well as hatching and fledgling success during the 1950s through 1970s when use of this pesticide was highest, with recovery in the following decades as DDT use was reduced and then banned in the UK, Europe, and North America^76^. Periods of low reproductive success and population declines were linked to reduced eggshell thickness, and therefore egg survival, that was observed in raptors shortly after use of this pesticide began in the UK^77^; this effect has since been found globally and linked to falcon population declines^78^. Lab studies confirmed that DDT’s metabolite DDE interferes with endocrine processes such as prostaglandin production that control calcium transport during eggshell formation^79^. In addition, male embryos of gull eggs injected with DDT had feminized reproductive tracts, which may explain historically low numbers of breeding males and skewed sex ratios in some populations^80^.

PFAS have also been found in elevated levels in seabird tissues globally, including species in remote regions like the Arctic and Antarctic^71^. A field study of a sentinel seabird species—black-legged kittiwakes (*Rissa tridactyla*)—in Svalbard (European Arctic) found positive correlations between abnormal sperm and plasma levels of some PFAS compounds^81^. Though there was little evidence of an underlying endocrine mechanism, with poor or no correlations between these individual PFAS and hormones (luteinizing hormone, testosterone, corticosterone), the increased presence of abnormal sperm is linked to reduced fertility in captive birds^82^. In addition, seabirds contain elevated levels of other contaminants that are known endocrine disrupters (e.g., PCBs, DDE^83^), and they may also be contributing to abnormal sperm cells^81^. In addition, climate change may exacerbate exposures and, therefore, effects, as elevated temperatures have been linked to greater plasma levels of organochlorine contaminants in seabirds^83^.

Birds, as apex consumers in terrestrial and aquatic food webs, are highly vulnerable to endocrine-disrupting contaminants and other stressors because of their tendency to bioaccumulate chemicals through diet and long lifespans, and their global exposures to numerous synthetic chemicals, which can act independently or in synergy to adversely impact population sizes. While DDT is the clearest example of exposures linked to reduced bird populations^76^, others, such as PFAS, warrant further mechanistic study given their links to abnormal sperm morphology and potential to reduce fertility. Notably, impacts on sperm from PFAS exposure are also documented in mammals, including humans^84^. Together, these studies underscore the vulnerability of birds to persistent and globally distributed contaminants in the context of climate change and emphasize the need for ongoing monitoring to assess avian population health.

### Reptiles

Sex chromosomes determine sex for some reptiles, but in most turtles and all crocodilians, sex is largely determined by temperature during development^85^. Chemical pollutants are now known to influence this process. In temperature-dependent sex determination (TSD), nest incubation temperature determines offspring sex ratios, with more of one sex predominating at incubation temperature extremes^85^. Temperature influences the developmental production of sex hormones, which then shape gonadal and behavioral sex. However, temperature shifts and extremes, and chemical pollution can perturb hormone signals and alter sex ratios and gonadal sex characteristics.

Reptiles with TSD are increasingly vulnerable to environmental factors. In many taxa, rising global temperatures skew sex ratios to favor females^86–88^. The foundational understanding of TSD occurred at about the same time changes in gonadal differentiation and sex-determination were being observed in wildlife exposed to chemical pollutants^89^. Convergence in understanding of climate-driven feminization with EDC-induced disruption of sex hormone signaling is now recognized as a probable threat to the viability of reptile populations reliant on balanced sex ratios.

Laboratory studies in red-eared slider turtles (*Trachemys scripta*) demonstrated that exogenous estrogens or anti-androgens could override effects of incubation temperature, causing sex reversal at male-producing temperatures^88^. This and subsequent work by Crews and colleagues described how temperature interacts with endocrine signals to “organize” sexual differentiation during critical periods of embryonic development to produce long-lasting effects on gonadal and neuroendocrine systems of reproduction^86,90^. Further research established that temperature and steroid hormones act synergistically to direct gonadal differentiation, underscoring the vulnerability of TSD species to disruptions of endocrine pathways^91^. In laboratory experiments, estrogenic PCBs applied to red-eared slider turtle eggs led to more hatchlings with female gonads at male-producing temperatures^92^. These studies revealed that even minor perturbations in hormonal actions during development permanently shift sexual phenotypes in individuals and populations.

Field studies of American alligators (*Alligator mississippiensis*) have provided compelling evidence of EDC impacts. Gonadal abnormalities were first documented, along with altered plasma sex steroid concentrations, in juvenile alligators from Lake Apopka, a lake contaminated with agricultural pesticides, including DDT^93^. Subsequent work revealed reduced plasma testosterone and smaller phallus size in males from Lake Apopka compared to lower contamination sites, yet no correlation between hormone levels and genital morphology was found in alligators post-embryonically^94^. These studies demonstrated that embryonic exposure to EDCs had permanently reprogrammed reproductive physiology during critical periods of embryonic development.

These findings were expanded by showing disrupted steroidogenesis in both field-exposed and experimentally-treated alligators^95^. Two major DDT metabolites, p,p′-DDE, which have anti-androgenic properties, and o,p’-DDE, which is estrogenic, were found in high concentrations in Lake Apopka alligators. American alligator eggs collected from a reference site and treated with 17-beta estradiol or a nonaromatizable androgen during embryonic development had changes in gene expression in juveniles consistent with those of juveniles reared from eggs collected from a DDT-contaminated site^96^. It was concluded that gonadal changes observed in wild alligators developing in contaminated lakes likely arose from environmental estrogens^96^. Others confirmed this further in field-collected alligator eggs^97^. Collectively, these findings represent key support for the now well-established hypothesis that synthetic chemical exposures adversely alter organizational effects of steroid hormones, and establish American alligators as a sentinel species for effects of EDCs on reproductive development.

Marine turtles, another TSD taxon, also show signs of synergistic impacts of climate change and chemical pollution^87^. Early field and subsequent laboratory studies demonstrated that nest temperature tightly controls sex ratios in sea turtle hatchlings^98–101^. Recent evidence from northern Australia found near-complete feminization of green sea turtle populations under warming conditions^102^, with a recent global synthesis documenting widespread female-biased sex ratios under increasing planetary temperatures^103^. Emerging evidence supports the conclusion that sea turtle populations, like freshwater turtles, face dual pressures from planetary warming and contaminant exposure, both of which can alter sex determination. Field studies document associations between trace metals, some of which are metallo-EDCs, and organic pollutants in hatchlings, likely leading to female-biased sex ratios^104^. These findings suggest that freshwater and sea turtles, like alligators, face compounded risks from elevated nest/incubation temperatures and exposure to EDCs and chemical pollutants.

The body of evidence from controlled laboratory manipulations, field-based alligator research, and sea turtle population studies highlights the convergence of climate warming and chemical pollution as dual threats to TSD reptiles. While climate change skews sex ratios toward feminization, EDCs disrupt androgen and estrogen signaling pathways to alter normal gonadal development, decreasing reproductive capacity and thus drastic population decline^89^. This dual stressor framework suggests that TSD species, while at imminent risk, may serve humanity as critical sentinels for understanding how global change processes intersect with legacy and emerging pollution, ultimately disrupting reproductive success and leading to population loss.

### Amphibians

Amphibians are the most threatened animal class on the planet. The declines are so severe that they may be part of a contemporary sixth major extinction^105^. By far the most studied amphibians are the frogs and toads (Anura), whereas studies on salamanders (Urodela/Caudata) and legless caecilians (Gymnophiona) are sparse. A substantial body of literature demonstrates the sensitivity of frogs and toads to conventional EDCs (e.g., atrazine, EE2, anti-androgens, pesticides, etc.; for review, see ref. ^3^). However, we are only just beginning to understand the impact of climate change-related impacts and non-conventional EDCs on these important sentinels.

As amphibian life stages often transition from one ecological niche to another, requiring different survival requirements (e.g., life on land versus aquatic life stages), the impact of climate change is especially poignant when one or more of these niches are compromised. For example, many amphibians require water for their larval stages and breeding. ^106,107^ Environmental conditions, such as early rainfall events or faster drying up of breeding habitat, can result in ultimately poorly timed or failed mating events. ^108^ Metamorphic timing and size impact reproductive capacity as well^106,107,109^. Ponds drying up faster than usual can result in an accelerated metamorphosis, leading to a smaller metamorph body size that can affect lifelong fitness. ^[,110^ For example, larger frogs with better body condition will have lower-pitched calls^111,110^, be more fecund^112^, have higher call rates^113^, and/or better-quality oocytes^112^. Moreover, as ectotherms, they are more sensitive to ambient temperature changes than endotherms like mammals, which can further impact body size/condition, sex differentiation, gamete performance, and/or mating behavior.

In contrast to reptiles, amphibians normally have autosomal-determined sex, independent of temperature. However, under certain conditions, high temperature can cause sex reversal in frogs, leading to male-biased sex ratios^114–118^. For example, wood frog (*Rana sylvatica*) tadpoles subjected to different holding temperatures in the fourth week post hatch at a time after sexual differentiation was complete, had a 50:50 sex ratio at 20 °C but were entirely male at 32 °C when examined at Gosner stage 42 at the onset of metamorphosis^115^. This is a result of a sex reversal event. No threshold temperature, as is often found in reptiles, was observed. Also, females metamorphosed larger and later in cooler conditions and smaller and earlier under warm conditions, suggesting that warmer conditions may impact female fitness.

In another study, Calebrese and Pfennig^117^ examined the effects of temperature on sexual signal production and related this to body size/condition, and breeding phenology of natural populations of spadefoot toads (*Spea multiplicata*). They found a complex relationship between changing temperature and male sexual signals, pointing to the need for more studies on the impacts of climate change on mating. They found that warmer breeding pond temperatures correlated with increased call and pulse rates, while call duration decreased. This suggests that there is a greater energy investment by males as temperatures increase. However, the researchers found no such relationship with body size or condition. It is noted that there was considerable variation in these data that could obscure a significant relationship.

The mechanism of temperature-dependent sex reversal in amphibians appears to be distinct from “conventional” endocrine disruption, as neither aromatase inhibitors^119^ nor environmentally relevant exposure of 17α-ethinyl estradiol (EE2)^114^ reduces the female-to-male sex reversing effects of high temperature. Epigenetic mechanisms involving sex differentiation are largely unknown in amphibians. However, histone acetylation changes in the *bod1l* gene promoter in tandem with male gonadal differentiation^120^. This, combined with the knowledge that histone methylation patterns are affected by temperature in frog tadpoles^121^ and that temperature impacts the gene expression response to conventional EDCs^122^ make epigenetic mechanisms an intriguing possibility.

Superimposed on the impacts of climate change are the impacts of microplastics (MPs) and other stressors, such as EDCs or toxins from algal blooms. In a recent review, over 80% of 33 studied amphibian species showed microplastic bioaccumulation^123^. As MPs impair tadpole body condition and function makes it highly plausible that reproduction could be affected^124^. While it is well-known that MP constituents like phthalates have androgenic activity by modulating steroid 5-reductase expression in Western clawed frog embryos^125^, MPs can also act as chemical pollutant carriers, particularly of hydrophobic chemicals that would have high affinity for plastics. For example, MPs sorb phthalates and BPA that are known to affect key reproductive endpoints in amphibians, including reproductive success, sex ratios, and spermatogenesis^126,127^. MPs also cause mechano-physical stress to amphibian eggs and affect hatching rates^123^. Together, the evidence is mounting that climate change and MPs can impact amphibian fertility both in concert with and independent of conventional EDCs.

### Marine mammals

Marine mammals are exposed to various environmental contaminants through dietary intake and the surrounding environments. As high-trophic-level organisms, marine mammals are particularly vulnerable to lipophilic synthetic contaminants that biomagnify through the food web. Studies across the globe have reported the occurrence of endocrine-disrupting persistent organic pollutants (POPs) in marine mammal tissues at levels in the parts per million (or milligram per kilogram) range based on tissue lipid weight^128–130^. These decade-long exposures have been linked to impacts on the endocrine system, leading to impaired reproduction, weakened immunity, and reduced survival of the fetus^131^. In recent years, researchers have also observed how climate-change-related stressors have exacerbated the effects of POPs on fecundity and fertility via endocrine and non-endocrine related pathways^132^. One group of marine mammals particularly affected are pinnipeds, including seals and sea lions that live in coastal habitats impacted by anthropogenic pollution and global warming. The case studies described below highlight the various stressors and pathways that have contributed to impaired reproductive success in seal and sea lion populations over the last few decades.

Pinnipeds are exposed to POPs via the fish they prey on, and levels measured in seal and sea lion samples often exceed species-specific toxicity thresholds^133,134^. Studies have also shown that pups are exposed to POPs through lactation^135,136^. The effects of POPs exposure have been observed at the molecular, organismal, and population-level effects. At the molecular level, PCBs and DDTs act as endocrine disruptors and alter reproductive and immune pathways. For example, increasing levels of PCBs are correlated with reduced levels of steroid hormones (i.e., estrogens, testosterone, progesterone, thyroxine) in harbor, ringed, and gray seals^137,138^. Studies that integrated experimental and epidemiological data have also revealed strong relationships between levels of PCBs and DDTs and the incidence of uterine pathologies, including tumors and occlusions, in Baltic gray seals^139,140^. This resulted in decreased pregnancy rates and a decline in populations. With the ban of PCBs and DDTs, tissue levels have decreased in recent years, and so has the incidence of uterine pathologies.

Seal and sea lion populations are also vulnerable to a group of naturally occurring chemicals called cyanotoxins. Under specific environmental conditions, cyanobacteria can form virulent blooms known as harmful algal blooms (HABs) during which high concentrations of cyanotoxins are released in the water column and can accumulate in fish and shellfish. Highly toxic cyanotoxins found in marine habitats include domoic acid and saxitoxins^141^. In the last decade, global warming has favored the formation of HABs, thus increasing their frequency and virulence and leading to severe impacts on pinnipeds ranging from neurological damage to deaths^142–144^. Exposure to domoic acid and saxitoxin has also been linked to decreased reproductive success. For example, Lefebvre et al.^144^ observed that cyanotoxins can be transferred from pregnant female sea lionizers to their fetus via fetal fluids. Other studies have shown that accumulation of domoic acid in pregnant females can also lead to preterm abortion or premature births and lower pup survival^145^. Although the specific reasons for the interrupted pregnancies are not well understood, these studies show that cyanotoxins can reduce fecundity, which could lead to population declines.

While synthetic chemicals are major contributors to endocrine disruption, other environmental factors can also negatively affect reproductive success. In the Arctic, global warming has impacted pinniped populations by reducing the habitats and food supply needed to successfully breed, birth, and rear their pups. The lack of food during the energy-demanding stage of pregnancy is believed to cause hormonal changes in pinnipeds, leading to increased pup mortality. Stenson et al.^146^ have shown a clear link between the loss of sea ice and the increase in late-term abortion and premature birth in harbor seals. In California, increased water temperature was identified as one of the factors affecting the immune functions of adult and pup sea lionizers, leading to reduced survival^147,148^. This growing body of evidence has highlighted the impacts of climate change on the pinniped endocrine and metabolic systems and the downstream effects at the population level.

### Rodents

Observations linking exposure to EDCs and adverse reproductive health outcomes in wildlife species and humans can be intensely and directly studied in laboratory animal models, most commonly laboratory rodents. The diversity of rodent models makes it possible for laboratory studies to unravel molecular pathways linking exposure to effects, across one or more compounds, at specific levels of exposure, across life stages, and/or with specific genetic pathways modified or absent. This makes them ideal models for deeper explorations into how EDCs and other stressors work at the molecular level.

Work with established EDCs such as BPA, phthalates, and flame retardants in rodent models has uncovered their ability to not only interfere with steroid hormone receptors and steroidogenesis directly, but to alter reproductive outcomes indirectly through impacts on physiological processes such as apoptosis, oxidative stress, and behaviors^149,150^. For example, a study by Susiarjo et al.^151^ demonstrated direct effects of BPA on reproductive tissues in mice. Fetuses exposed to BPA via dams during mid-gestation had disturbed oocyte development in ovaries that translated into increased chromosomally abnormal eggs and embryos when the fetuses reached adulthood. The same study also demonstrated that these oocyte disturbances arose from BPA interaction with an estrogen receptor^151^. Other work showed indirect effects of developmental phthalate exposure on reproductive endpoints in mice. In adult offspring treated with a phthalate during gestation and lactation, numerous behaviors related to sexual success were impaired, including mate attraction, olfaction, and copulatory behaviors^152^. Thus, studies of legacy EDCs in experimental rodent models have demonstrated that they impair reproduction through myriad mechanisms ranging from direct effects on reproductive tissues, hormone signaling, and hormone production to indirect effects from impacts on related physiological processes. These rodent-based studies can inform future research with emerging or understudied synthetic chemicals and can guide epidemiological study endpoints to uncover or verify effects of EDCs on human reproductive success (or failure). Interestingly, some of the foundational work underscoring studies with EDCs in rodents didn’t concern exogenous chemicals at all.

It can be challenging to conduct studies on emerging and combined environmental stressors, such as synthetic chemicals or temperature extremes, in laboratory animal models. Often, very little information is available on “relevant” doses, routes of exposure, duration of stressor events, and how these are translatable to wildlife and/or human exposure conditions. Similarly, effects-directed studies may only be launched after evidence of such effects are uncovered from ecological or epidemiological studies. Although some studies with laboratory animal models may lag behind environmental discoveries, they are critically important for those deeper dives into mechanistic pathways and for establishing dose-response relationships that support public and environmental health protective measures.

Some experimental model studies with emerging chemicals, however, may identify public and environmental health concerns before they are observed in ecological or epidemiological studies. Early work with the legacy PFAS, PFOA, revealed effects on reproductive and developmental endpoints before these were explored in, for example, exposed human populations. White et al.^153^ described altered mammary gland development in mouse dams and their offspring exposed to PFOA from gestational days 1 through 17, and in a follow-up study, they determined that this change persisted through three generations^154^. Similarly, studies of MNP in rodent models have evaluated individual polymers separately and in mixtures to identify potential human health concerns and mechanisms of action. As reported in a review by Da Silva et al.^23^, MNP can accumulate in reproductive tissues, cross the placental barrier, induce changes in germ cells in both female and male tissues, and alter hormone levels and cellular signaling pathways critical for reproductive function. MNP maternal exposures can also induce placental toxicity and fetal growth restriction via alteration of gene expression in both the placenta and the fetus^155^. This study is especially concerning as it demonstrates that these “inert” toxicants are able to interact with multiple receptors to affect cellular functions. Coffin et al.^20^ summarized available studies to derive a human health screening level for MNP in California drinking water. Of the 12 mammalian studies identified, more than half of the studies reported adverse effects in the reproductive systems of both sexes, including hormone disruption and changes in sperm count, mobility, structure, and viability^20^. These types of studies in laboratory animal models are therefore essential for creating foundational knowledge, alerting the ecological and epidemiological communities to relevant endpoints to evaluate, identifying molecular mechanisms of toxicity, and informing public health efforts.

Emerging chemicals can also be compared and contrasted to more well-studied chemicals to evaluate potency, as demonstrated by ref. ^156^ (2019) in a comparison of estrogenic effects of hexafluoropropylene oxide (HFPO) homologs with PFOA. Xin et al.^156^ evaluated estrogenic effects of HFPOs compared to PFOA with ligand binding, transcriptional activity, and in vivo assays with zebrafish and observed that HFPO compounds, which were intended to replace PFOA in certain applications, have more potent estrogenic effects than PFOA. These types of comparative studies in laboratory animal models can be especially valuable in deriving relative potency factors for chemicals within the same class for use in site- or media-specific risk assessments.

### Humans

A key characteristic of EDCs is their ability to alter hormonal signaling. Reproductive development depends heavily on signaling (particularly) of the steroid hormones, receptor binding, and metabolic action. Here we discuss three examples of disruption of human reproductive health by EDC exposure: (1) the phthalate syndrome; (2) MPs and male reproductive health, and (3) impacts of PFAS and pesticide exposure on fertility.

To begin, phthalates are high-production chemicals used primarily as plasticizers in polyvinylchloride. Widespread human phthalate exposure in the US was first documented in 1999 by the National Health Examination Survey (III)^157^. In 2000, scientists at the EPA identified the “phthalate syndrome”, a collection of male reproductive abnormalities caused by prenatal exposure to the most anti-androgenic phthalates. This syndrome in males, resulting from a phthalate-induced decrease in testicular testosterone production in early pregnancy, includes hypospadias, cryptorchidism, nipple retention, and a reduced anogenital distance (AGD)^158^. It was later shown that the phthalate syndrome is produced by prenatal exposure during a critical developmental window (termed the male programming window, or MPW)^159^.

The most notable symptom of the phthalate syndrome is shortening of the AGD, which, in most mammals, is 50–100% longer in males than in females. Initially, a human analog of the “phthalate syndrome”, which was associated with the same anti-androgenic phthalates (diethylhexyl phthalate, butylbenzyl phthalate, and dibutyl phthalate) was found, which caused the phthalate syndrome in rodents^160^. The phthalate syndrome was subsequently confirmed in humans in two European cohorts^161,162^. The implications of short male AGD on reproductive health remained unclear until Eisenberg et al. showed that AGD was shorter in infertile men^163^ and Mendiola and colleagues demonstrated a significant correlation between AGD and sperm count in adulthood^164^.

Pesticides, including those that are currently commonly used in and around homes, such as pyrethroids, also have impacts on reproductive parameters^29^. A recent scoping review found consistent adverse associations between pesticide exposure and reduced sperm quality, particularly sperm motility, DNA integrity, concentration, and morphology, aligning with earlier systematic reviews^165^. Supporting this, Meeker et al.^166^ reported that higher urinary pyrethroid metabolite levels were linked to reduced semen quality and increased sperm DNA damage in men from an infertility clinic^167^.

Another contaminant class of concern with a high incidence of human exposure are the PFAS chemicals, which interfere with a wide range of hormones, including sex steroids, thyroid, and metabolic hormones in humans. Studies demonstrate significant associations between PFAS exposure and fertility in both men and women. For example, the Singapore Preconception Study of Long-Term Maternal and Child Outcomes measured PFAS in 382 women trying to conceive. The authors found fecundability significantly decreased with increasing exposure to PFAS chemicals individually, and in combination^168^.

Shen and colleagues studied the associations between in vitro fertilization (IVF) outcomes and plasma concentrations of 8 individual PFAS and PFAS mixtures in 259 women undergoing in vitro fertilization and embryo transfer (IVF-ET). This study demonstrated that exposure to PFAS chemicals, individually and in mixtures, negatively affects oocyte yield, fertilization, and embryo quality in women undergoing IVF^169^. A recent systematic review summarizes the literature published 2017–2022 on PFAS in relation to fertility and other reproductive outcomes in the general population. This review, which included 30 eligible studies (with a total of 27,901 participants), found no effect of background levels of PFAS on fertility overall. However, PFAS moderately increased the odds of PCOS- and endometriosis-related infertility. This study also found that sperm motility and DNA health were moderately impaired by multiple PFAS^170^.

More recently, concerns have arisen regarding the reproductive impacts of MNPs. Adverse effects of MNPs on male reproductive function were first reported in rodent studies in 2021. These earlier studies, mostly focused on male reproduction and polystyrene MPs, reported impacts on sperm count and hormone levels^20,171^. Subsequent studies examined these associations in humans and canines. In 2023, a study reported MPs in 5 human testes and 30 human semen samples, raising concerns about their potential accumulation in the male reproductive system^21^. A second study reported the presence of microplastics in all (47) canine and (23) human testes, with total MP concentrations considerably higher in humans than in dogs. Both humans and canines exhibit similar proportions of polymer types, with PE being dominant. This study documents the pervasive presence of MPs in both canine and human testes, with potential consequences on male fertility^171^. Several studies have examined the joint effects of a mixture of MPs in human samples. Zhang et al.^172^ detected 3–5 different types of microplastics in all semen and urine samples tested, with the highest detection rates for polystyrene, polypropylene, PE, and polytetrafluoroethylene. All types were significantly associated with decreases in total sperm number, concentration, and progressive motility^158^.

At the population level, there is epidemiological evidence that exposure to EDCs can influence the ratio of male to female live births in humans. Many of the studies that have examined these exposures have followed notable contamination episodes. In most cases, the sex ratio is decreased following prenatal exposure. In some instances, stronger associations are seen following paternal exposure, and exposure occurring in adolescence or young adulthood, consistent with impacts on spermatogenesis. Chemicals most frequently associated include the historic exposures to dioxin, PCBs, and DBCP. In addition to chemical exposure, as with other taxonomic groups, humans can be impacted by increasing temperatures. An analysis over 80 years, using birth data from the U.S., shows that hotter weather is associated with reduced conception rates^173^. Higher ambient temperature also negatively affects semen volume, sperm count, motility, and morphology^174^. In summary, these studies illustrate the reproductive harms caused by human exposure to phthalates, microplastics, and PFAS, as well as higher temperatures. Each is an example of a reproductive toxicant that, individually and (usually with greater impacts) as mixtures have reproductive consequences. These adverse effects are not limited to humans but can be seen, as discussed above, across multiple species.

## Conclusion

Strong cross-species evidence demonstrates that exposure to a diversity of environmental stressors is linked to profound reproductive impacts in invertebrates, fish, terrestrial wildlife, rodent models, and humans. These findings underscore the urgent need for regulatory and scientific frameworks that address entire classes of chemicals known to disrupt hormonal systems across multiple taxa, rather than focusing on individual compounds. Historical cases illustrate both the power and limitations of regulatory action. For example, European nations began restricting TBT in the early 1980s, while in the U.S., congressional legislation in 1988, prompted by expert warnings of marine toxicity, bypassed usual federal processes and directly restricted TBT-based paints, leading to declines in environmental concentrations and related improvements in species’ health^41^^,^^42^. Similar successes can be seen in the regulation of DDT and PCBs under the Stockholm Convention, which markedly reduced global exposures, though uneven enforcement and gaps outside Europe and North America are still problematic.

The urgency of current negotiations toward a Global Plastics Treaty reflects recognition that plastic pollution—carrying thousands of potential EDCs and other stressors—represents not only an ecological but also a planetary health crisis, as demonstrated by the data presented herein^2,175,176^. Ecosystem and human health are deeply interconnected: warming temperatures, hypoxia, and chemical exposures interact to exacerbate reproductive stress. Human fertility trends, including sex ratio shifts following natural and anthropogenic disasters, parallel wildlife responses and highlight that all living organisms are involuntarily exposed to chemicals that have not been thoroughly vetted for safety.

Compounding these issues are the complexities of exposure routes, from dietary and environmental uptake to habitat-dependent differences. Combinations of abiotic stressors, habitat degradation, and multiple chemical classes often amplify reproductive toxicity. Evidence from multigenerational and transgenerational studies^177^ demonstrates that the impacts of EDCs may be worse in the offspring of exposed parents, raising concerns about the persistence of reproductive harm long after exposures occur. Unlike humans, who may attempt to “choose” or delay reproduction under stress, invertebrates, fish, and wildlife have no such agency, magnifying risks of biodiversity loss and population decline.

Taken together, these examples emphasize that a diverse array of pollutants, combined with increasing pressure from worsening climate change, threaten fecundity, fertility, biodiversity, and health on a global scale. Addressing them requires coordinated, transboundary regulatory action.

## Data Availability

No datasets were generated or analyzed during the current study.
